# Long-Term Functional Outcomes and Complications of Intra-Articular (AO type B, C) Distal Humerus Fractures in Adults: A Retrospective Review

**DOI:** 10.7759/cureus.21094

**Published:** 2022-01-10

**Authors:** Minos Tyllianakis, Konstantina Solou, John Lakoumentas, Andreas Panagopoulos

**Affiliations:** 1 Orthopaedic Department, Medical School, University of Patras, Patras, GRC; 2 Medical Physics Department, Medical School, University of Patras, Patras, GRC

**Keywords:** ao type b-c, long-term follow up, complications, internal fixation, intra-articular fractures, distal humerus

## Abstract

Introduction

Treatment of intra-articular fractures of the distal humerus is challenging due to their complexity, comminution, and associated complications. The evolution of surgical approaches and the design of elbow-specific implants over the last decades have failed to improve clinical and radiological outcomes. Studies are sparse regarding the long-term influence of surgical treatment of these types of fractures in the upper limb function. The purpose of the current study was to retrospectively review the long-term functional outcome and complications of all intraarticular (AO type B, C) distal humerus fractures treated surgically in a university hospital during the last 25 years.

Material and methods

The study included patients who were surgically treated for an intra-articular distal humerus fracture between March 1991 and May 2016. Our initial search, using ICD-10 codes, identified 63 patients in the specific time period. Twelve patients had died, nine declined to participate, eight had emigrated, nine could not be located and one patient was excluded as he suffered from quadriplegia unrelated to the initial injury. The remaining 25 patients (mean age at surgery 44.2±19.67) were included in a follow-up study protocol. Functional outcome was evaluated according to Mayo Elbow Performance Score (MEPS), Oxford Elbow Score (OES), and the Quick Disabilities of the Arm, Shoulder and Hand questionnaire (QuickDASH). Pain was assessed using the Numerical Pain Rating Scale (NPRS) and subjects were asked to rate their satisfaction. Perioperative and late complications were recorded as well.

Results

The average follow-up was 158.16 ± 73.73 months. The average score was 89.4 ± 12.36 for MEPS, 42.68 ± 4.03 for OES, and 8.1 ± 15.38 for the QuickDASH. The patient satisfaction was rated 3.8 ± 1.08 on average. The mean flexion of the affected elbow was 137.6 ± 12.68 degrees while extension deficit was present in 14/25 patients, with a mean of 8.6 ± 8.96 degrees. We did not observe any mal- or non-unions. The re-operation rate was 32% primarily due to stiffness and irritation from the hardware. We noticed one spontaneously resolved ulnar nerve palsy, one combined radial, and ulnar nerve palsy after extensive arthrolysis that also resolved two cases of heterotopic ossification, one case of implant failure, and two cases of infection - one superficial, which was managed with antibiotics, and the other was deep managed with surgical drainage.

Conclusions

In our series, we found a satisfying range of motion, good functional outcome, and adequate ability to perform daily activities at a very long follow-up. Posttraumatic arthritis, whenever present, does not seriously affect functional performance.

Level of evidence: IV

## Introduction

The overall incidence of distal humeral fractures in adults has been estimated to be 5.7 cases per 100,000 person-years, with an almost equal male to female ratio and a bimodal age distribution with one peak in young males and a second peak in elderly females [1]. More recent epidemiological studies have shown increased risk in older patients (83% > 50 years) due to a simple or an unspecified fall (79% > 50 years) with a female/male ratio of 2.4:1; among them 59.2% were specified as intra-articular according to AO/OTA classification (type B: 25.1%, type C:34.1%) [2-3].

AO type B and C fractures are considered unstable due to their intra-articular extension and/or comminution and require meticulous surgical intervention. In reality, a proper anatomical reduction and adequate internal fixation can become a real challenge [4-6]. Despite the evolution of surgical approaches and design of elbow-specific implants in the last decades, recent studies have failed to show significantly improved outcomes [7-11]. Malunion, nonunion, deformity, stiffness, heterotopic ossification, and complications from the olecranon osteotomy and the ulnar nerve are consistently reported and are mainly related to cartilage loss, inadequate reduction, and suboptimal fixation [4, 12-15]. Yetter et al. reported recently in their systematic review and meta-analysis a total complication and reoperation rate of 53.4% and 20.8% respectively [15]. Nevertheless, malunion is not necessarily correlated always with unsatisfactory results [16,17]. To our knowledge, few studies have presented long-term results of such fractures [18-21]. The purpose of this study was to retrospectively review the long-term functional outcome and complications of all available patients with intra-articular distal humerus fractures, treated surgically in our university hospital in the last 25 years.

## Materials and methods

Study population

All patients that sustained an intra-articular distal humeral fracture between March 1991 and April 2016 were identified from the electronic database of our Orthopaedic Department. The exclusion criteria were extra-articular fractures (AO/OTA type A), ipsilateral fractures of distal humerus and forearm (floating elbow), preexisting deformity, stiffness or neurological deficit in the ipsilateral upper limb, and previous surgery to the same elbow. Our initial search identified 63 patients treated for an intra-articular distal humerus fracture (AO/OTA type B, C) in the study period. Patients were treated by two experienced orthopaedic surgeons. Twelve patients had died, nine declined to participate, eight had emigrated, nine could not be located and one patient had been excluded due to quadriplegia unrelated to the initial injury at the final follow-up. The remaining 25 patients were included in a follow-up study protocol that had been approved by our institutional review board with approval no. 144/06.06.2020/ΑΔΑ6ΝΩ346906Γ-Φ6Ω.

Patient characteristics

Twenty-five patients were re-examined clinically and radiographically between January 2020 and March 2021, at a mean of 158.16±73.73 (48-305) months after the initial treatment. Open reduction and internal fixation were performed at an average of 2.4 days (1-6 days) after the injury and the mean hospital stay was 4.8 days (4-35). There were eight patients (32%) with concomitant injuries in other locations requiring prolonged hospitalization. The patients were routinely re-examined at three, six, and twelve months postoperatively. The raw data including a full demographic profile, patient age, sex, and a standardized radiological workup was collected from the Hospital’s files.

Fracture classification

Two of the authors (AP, KS) reviewed independently the primary radiographs to classify the fractures according to AO/OTA classification [3] and to evaluate implant position, heterotopic ossification and evidence of posttraumatic arthritis. There were six type B (2 B1, 1 B2, 3 B3) and 19 type C fractures (1 C1, 12 C2 and 6 C3).

Surgical technique

All patients were operated under general anesthesia in the prone or lateral decubitus position with the involved arm hanging over a radiolucent arm support. High arm tourniquet inflated to 250-280 mmHg was used in all cases and three doses of a second-generation cephalosporine (Ceforanide) for antibiotic prophylaxis. After proper arm preparation and draping, a standard posterior midline approach was used with ulnar nerve preparation, identification, and protection. A V-type (chevron) osteotomy of the olecranon with a proximal reflection of the osteotomized olecranon and the extensor apparatus was used in 21 cases, whereas a lateral Koher approach was used in other four cases. Open reduction and internal fixation (ORIF) were achieved with headless screws, K-wires, or plates (3.5mm DCP, 3.5mm LCP, one-third tubular plates, or 3.5mm pelvic reconstruction plates, at 90° or 0°). Double plating was used in 11 cases. Anterior ulnar nerve transposition was not performed unless the nerve was in contact with implants at the end of surgery (n=8). Olecranon osteotomy was fixed with the tension band technique (n=16) using two 2.00mm K-wires or a 6.5mm cancellous lag screw (n=4). Soft tissues were closed in layers. A suction drain and an above-elbow removable cast were applied in all patients. Passive flexion and extension were commenced on the second or third postoperative day as pain allowed. The removable cast was discontinued after two to three weeks depending on the fracture pattern, stability of fixation, and the patient’s cooperation. A program of extensive active physiotherapy of the elbow was then initiated. At the final follow-up, the patients were either assessed in the outpatient department of our hospital by an independent examiner (KS) not involved in the initial treatment or by other orthopedic surgeons in the patient’s residential area since transfer was difficult due to Covid-19 imposed restrictions. Data of the latter was sent to us later. A detailed clinical examination including range of motion (ROM) in both elbows, pain at fracture site, and elbow stability were recorded.

Outcome assessment

I. Patient-Reported Outcome Measurements (PROMs)

All patients completed the Greek version of the QuickDASH score and the original versions of the Mayo Elbow Performance Score (MEPS) and the Oxford Elbow Score (OES) questionnaires. As no formal Greek translations exists for the latter two scores, these were administered by an independent investigator (KS) and explained to the patients. Patients were also asked to classify their pain on a scale from 0 to 10, where 0 indicated no pain and 10 the worst pain, according to the Numeric Pain Rating Scale (NPRS). We also examined the relationship between the score on each of the outcome instruments (MEPS, OES, DASH) with six demographic and clinical variables: age, gender, AO/OTA classification (B or C), follow-up time, ROM, and pain.

II. Satisfaction, Complications, and Radiological Evaluation

At the final follow up, satisfaction was assessed and categorized in terms of pain relief, functional performance in daily activities and recreational activities, in five groups as: very satisfied (5), satisfied (4), somewhat satisfied (3), unsatisfied (2), disappointed (1). Moreover, all post-operative complications and reoperations were recorded. No intraoperative complication was reported.

Statistical analysis

All quantitative variables were examined for normality with the Shapiro-Wilk test, and most of them were found to be non-normal. Therefore, non-parametric statistical tests were used in order to identify correlations or associations. Quantitative-quantitative correlations were assessed with Spearman’s rho correlation, while quantitative-qualitative associations were assessed with Wilcoxon rank-sum test. Regarding the descriptive statistics, mean ± standard deviation was used for quantitative variables, while count (%) for qualitative variables. All of the statistical tests were two-sided, and statistical significance was considered when p<0.05. The statistical analysis was performed in R, the language for statistical computing, along with the use of the RStudio IDE (RStudio, PBC, Boston, MA), both of which are open-source products.

## Results

Twenty-five patients were available at the final follow-up evaluation, at a mean of 158.16±73.73 (48-305) months after the index operation (Table 1). Both injured and uninjured sides were evaluated. There were eight males and 17 females with a mean age of 44.2±19.67 (18-82) years at the time of injury; the mean age at the time of follow-up was 57.48±18.3 (29-96) years. A simple fall on the elbow from a standing height was the most common mode of trauma (n=19), followed by road traffic accidents (n=5) and one fall from a substantial height. Eight patients (32%) had associated injuries elsewhere, including three polytrauma patients; one patient had a Gustilo I open fracture. At the time of injury, 11 patients were employed as laborers, four were white-collar workers, two were students, and eight were homemakers. The fractures occurred in 15 right and 10 left elbows. Open reduction and internal fixation were performed at an average of 2.4 days (one to six days) after the injury and the mean hospital stay was 4.8 days (4-35).

**Table 1:** Demographic data, range of motion, clinical outcome, complications and reoperations of the included patients. M – Male, F – Female, NPRS – Numeric Pain Rating Scale, MEPS – Mayo Elbow Performance Score, OES – Oxford Elbow Score, HO-Heterotopic Ossification, n/a – non applicable

Case number	Age (surgery)	Age (fup)	Sex	AO	Follow up time (months)	Flexion (degrees)	Extension’s deficit (degrees)	NPRS	MEPS	OES	QUICK DASH	Satisfaction	Complications	Posttraumatic Arthritis (grade 0 to 3)	Reoperation (n)
1	28	36	M	C2	95	140	0	1	100	48	0.0	3		n/a	
2	39	61	F	B3	263	140	0	1	95	46	2.3	5		n/a	
3	52	60	F	C3	89	140	0	1	95	44	2.3	5		n/a	
4	72	88	F	C2	186	140	0	1	95	45	2.3	4		1	
5	71	80	F	C2	119	140	20	4	80	34	20.5	4		3	
6	62	66	F	C3	48	140	20	7	80	32	11.4	4		3	
7	60	64	F	B3	48	110	10	1	95	43	6.8	2	Pain-stiffness	2	1
8	40	52	F	C2	141	140	10	1	95	45	2.3	4		n/a	
9	24	50	M	C3	305	150	0	1	95	44	0.0	2	Pain-stiffness	1	1
10	64	80	F	C2	187	150	0	4	95	41	18.2	4	Pain-stiffness	n/a	1
11	82	96	M	C2	163	140	0	3	55	43	75.0	4		2	
12	45	54	F	B1	108	140	0	4	85	43	2.3	4		n/a	
13	40	59	F	C2	229	140	0	1	95	45	6.8	5		n/a	
14	53	59	M	B2	66	150	10	9	55	33	18.2	5	Pain-stiffness	3	1
15	21	29	F	C1	107	140	15	1	100	45	0.0	5		n/a	
16	27	41	M	C2	176	140	20	2	85	44	4.5	2	Deep infection	2	1
17	27	41	F	C2	175	140	10	1	100	45	0.0	5		0	
18	18	33	M	C2	176	140	10	1	100	45	0.0	2	Superficial infection	1	
19	54	74	F	C3	240	150	0	1	85	41	13.6	4		n/a	
20	18	39	M	C2	254	140	10	1	100	45	0.0	4		0	
21	18	33	M	C3	171	100	20	1	95	45	0.0	4	Pain-stiffness	n/a	1
22	31	44	F	C3	154	150	0	1	85	44	0.0	3		n/a	
23	74	81	F	B3	83	130	30	2	80	41	11.4	2	Stiffness (HO)	n/a	1
24	33	57	F	C2	282	140	15	0	100	45	0.0	4	Resolved ulnar nerve palsy	0	
25	52	60	F	B1	89	110	15	1	90	41	4.5	5	Hardware failure, Resolved radial and ulnar nerve palsies	2	3

All patients presented with a normal ROM on the unaffected elbow. The mean range of flexion of the affected elbow was 137.60±12.68 (100-150) degrees and the mean extension deficit 8.60±8.96 (0-30) degrees. Three patients (Nos. 7, 21, 25 respectively) had severe restriction of elbow motion (flexion ≤ 110° and extension deficit 10°, 15°, and 20° respectively); all of them had undergone surgical release and implant removal at a mean of 6 to 16 months after the initial operation (Table 2).

**Table 2:** Clinical outcome of the included patients fup – follow up period, F – Female, M – Male, NPRS – Numeric Pain Rating Scale, MEPS – Mayo Elbow Performance Score, OES – Oxford Elbow Score

Variable	Descriptives *
Age (surgery)	44.20 ± 19.67 (18 - 82)
Age (fup)	57.48 ± 18.30 (29 - 96)
Sex	
F	17 (68%)
M	8 (32%)
AO type	
B	6 (24%)
C	19 (76%)
Follow up time (months)	158.16 ± 73.73 (48 - 305)
Flexion (degrees)	137.60 ± 12.68 (100 - 150)
Extension's deficit (degrees)	8.60 ± 8.96 (0 - 30)
NPRS	2.04 ± 2.11 (0 - 9)
MEPS	89.4 ± 12.36 (55 - 100)
OES	42.68 ± 4.03 (32 - 48)
QUICK DASH	8.10 ± 15.38 (0 - 75)
Satisfaction	3.65±1.06 (2-5)
quantitative variables: mean ± SD (min - max), qualitative variables: count (%)	

The mean MEPS score was 89.4±12.36 (55-100) and in 16 patients the results scored as “excellent” while seven patients achieved a “good” result and two had “poor”. One of the latter (No=11) was bedridden (96 years old) while the other one (No=14) was a builder who reported chronic pain. The mean OES was 42.68±4.03 (32-48); 22 out of 25 patients rated between 40 to 48 and had a satisfactory function without clinical evidence of severe posttraumatic arthritis. As we didn’t obtain radiological evaluation in all patients due to their refusal or inability to attend our outpatient department, we were not able to correlate the values of OES with the degree of arthritis. In three patients, however (Nos. 5, 6, and 14 respectively), with very low OES (34, 32, and 33 in respect) the radiological evaluation revealed severe arthritis (stage 3 according to Broberg and Morrey classification). The mean QuickDASH Score was 8.10±15.38 (0-75). Fourteen patients achieved an “excellent” score, four “good”, and six patients had a “satisfactory” outcome. Only one patient (the bedridden one) had a poor result. Finally, in the NPRS evaluation, only two patients had severe pain; patient No. 14 had serious pain (9/10), despite a secondary surgical treatment for implant removal and joint arthrolysis one year after the initial operation; his radiological evaluation revealed a grade 3 posttraumatic arthritis, but he refused any further treatment. Patients were in general satisfied with the treatment having a mean satisfaction score of 4.06 ± 0.97 for the female patients and 3.25 ± 1.16 for the male patients. Five patients (20%) were unsatisfied with the final outcome.

There was no statistical significant correlation of sex, type of fracture, length of follow up and ROM with any of the PROMS; only OES had a strong association with the length of follow-up, probably as it is a specific score for the detection of elbow arthritis. The parameters of age, both at the index procedure and at follow up and the NPRS had statistical significant correlation with the clinical scores (Table 3). 

**Table 3:** Inferential analysis of different variables with clinical scores fup – follow up period, F – Female, M – Male, NPRS – Numeric Pain Rating Scale, MEPS – Mayo Elbow Performance Score, OES – Oxford Elbow Score

Variable	MEPS		OES		QUICK DASH	
	descriptives *	p-value	descriptives *	p-value	descriptives *	p-value
Age (surgery)	-66.37%	<0.001	-65.13%	<0.001	84.46%	<0.001
Age (fup)	-60.09%	0,001	-57.83%	0,002	80.59%	<0.001
Sex		0,833		0,418		0,353
F	91.18 ± 7.19		42.35 ± 3.92		6.16 ± 6.57	
M	85.62 ± 19.54		43.38 ± 4.44		12.21 ± 26.14	
AO		0,107		0,141		0,180
B	83.33 ± 15.06		41.17 ± 4.40		7.58 ± 6.22	
C	91.32 ± 11.16		43.16 ± 3.91		8.26 ± 17.45	
Follow up time (months)	39.09%	0,053	46.11%	0,020	-30.41%	0,139
Flexion (degrees)	-12.17%	0,562	-16.09%	0,442	7.62%	0,717
Extension's deficit (degrees)	-11.70%	0,577	-23.38%	0,261	4.39%	0,835
NPRS	-73.89%	<0.001	-71.77%	<0.001	70.11%	<0.001
* quantitative targets: Spearman's correlation coefficient, qualitative targets: mean ± SD per target level						

The overall rate of postoperative complications was 40% (10/25 patients); six of them demonstrated postoperative stiffness and underwent implant removal and arthrolysis. Symptoms were resolved and ROM improved in four of them; one patient was not improved but declined any further treatment and another one with severe heterotopic ossification was partially improved. There was one early superficial infection managed with intravenous antibiotics and one late deep infection (nine weeks postoperatively) treated with surgical lavage and implant removal as the fracture was already healed. One patient developed postoperatively ulnar nerve palsy that was resolved spontaneously six months later. Finally, one patient (No. 25) with a coronal shear fracture (B1) demonstrated implant failure (protrusion of a Hebert screw) and underwent removal; six months later she had compromised ROM and underwent extensive arthrolysis that improved motion but was complicated with ulnar and radial nerve palsies that resolved nine months later. At her last follow up she had a reasonable ROM 110-15 degrees, good PROMS, and grade 2 arthritis. The overall reoperation rate was 32% (8/25 patients). In seven patients, the K-wires of the olecranon osteotomy fixation were removed under local anesthesia.

**Figure 1:**
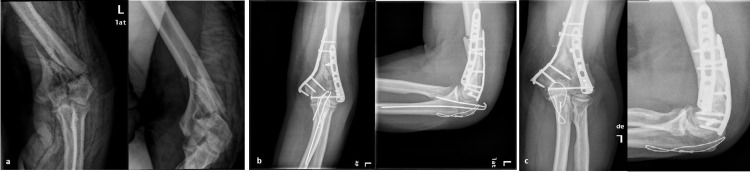
A case of type C intra-articular fracture (a) preoperative anteroposterior and lateral radiograph of an intraarticular fracture of the distal humerus (patient No 6), (b) one-month postoperative radiograph showing adequate reduction with the use of double plating, (c) late follow-up radiograph showing grade 3 post-traumatic arthritis; these patients had also removal of KW under local anesthesia.

We managed to obtain radiographs from only 13/25 patients, because of the limited movement of people due to Covid-19 or their reluctance to undergo radiological examination. In three of them (No = 5, 6, and 14 respectively), were depicted signs of severe posttraumatic arthritis (grade 3). They had moderate to severe elbow pain and scored low in all PROMS. Two of these patients (Νο. 5 and 14) firmly refused any further surgical intervention. The other (Νο. 6) had been examined by a private orthopaedic doctor and it is unknown what has been discussed (Figure 1). Four patients (Nos. 7, 11, 16, and 25 respectively) demonstrated grade 2 arthritis but they had a good clinical outcome except one. The remaining six patients had almost normal radiographs (grade 0 or 1) and all of them had excellent PROMS.

## Discussion

Open reduction and internal fixation remain the gold standard treatment of intraarticular fractures of the distal humerus with the restoration of the articular surface being paramount [7, 22]. Factors contributing to the complexity of these fractures are the small dimension of fragments which can limit screw fixation and osteoporosis requiring implants providing good bone purchase [12, 16, 23, 24]. Despite advances in implant design, several retrospective studies still present high complication rates, moderate functional results, and high revision rates [9, 14, 25]. Yetter et al. reported in their systematic review and meta-analysis (83 studies) a total complication and reoperation rate of 53.4% and 20.8% in respect among 2362 elbows (83% type C and 17% type B) and follow-up times to a mean period of 2.6 years [15]. The most common complication was implant irritation, malfunction or discomfort (10.1%), followed by ulnar neuropathy (8.2%), osteoarthritis (7.8%), elbow stiffness/contracture (7.5%), heterotopic ossification (5.1%) and infection (4.4%). Implant removal (11.4%), revision ORIF or total elbow arthroplasty (3.5%), and surgical release (2.8%) were the most commonly reported reoperations [15].

In our study, elbow ROM was not significantly restricted having a mean flexion of 137.60 degrees while the extension was restricted in 14 patients with a mean extension deficit of 8.60 degrees. The ability of patients to perform daily tasks with the injured elbow was rated by MEPS with a mean 89.4% of the maximal possible value. Moreover, elbow pain, elbow function, and social-psychological effects were assessed by a patient-focused outcome measure OES, with a mean value of 42.68. QuickDASH score was performed to evaluate an individual’s ability to complete tasks, absorb forces, and the severity of symptoms, and was rated with a mean of 8.10. These results suggest that most patients had a good outcome and almost normal elbow function in daily living activities.

Comparing our results with ones presented in previous long-term studies of similar fractures, Elhage et al. reviewed 55 patients, showed that 70.9% of the patients had above 120° flexion and 7.27% had less than 100° of flexion, in comparison with 91.3% of patients with flexion above 140° and 8.6% with flexion less than 110° in our study. Their cohort had 38.38% of the patients with >30° extension deficit, 40% with deficit 15°-20°, and 21.81% with deficit < 15°. The mean MEPS rated 78.8% in contrast to 89.4% in our study [18]. In a previous study from our department with a mean follow-up of 70.2 months in 26 patients, 23.1% had excellent results according to Morrey score, 57.6% very good and 19.3% fair. Besides elbow stiffness, infection, and ulnar nerve palsy, other complications reported were K-wire migration (n=3), scar breakdown (n=1), instrumentation failure (n=1), and heterotopic ossification (n=3). The overall re-operation rate was reported as 38.4%, compared to 32% of our study [19].

Doornberg et al. reported the long-term results of 30 patients with type C fractures in a mean follow-up period of 18 years (12-30 years). The average final flexion arc was 106°, the average (DASH) score was 7 points, and the average M EPS 91 points. Including one patient treated with elbow arthrodesis, the final categorical ratings were 19 excellent results, seven good results, one fair result, and three poor results. Interestingly, the presence of arthrosis did not appear to correlate with pain or predict disability or function. Subsequent procedures were performed in twelve patients (40%) [20]. Finally, Saragaglia et al. reported on 74 patients (33 type A, 6 type B, 35 type C fractures) treated with an inverted Y shape plate (Lambda® plate, Zimmer, Étupes, France) and evaluated after a mean follow-up of 115±64 months [21]. Mean elbow ROM exceeded 100° in 58 patients (77%), was between 50° and 100° in 16 (21%), and was less than 50° in one. Mean MEPS was 97±7 points and mean QuickDASH was 10 ± 18. There were 67 excellent results, five good, two moderate, and one poor, without significant difference in the group of 35 type-C fractures. They also reported one case of non-union of the lateral condyle and one of the distal part of the humerus, two cases of dysesthesia in the ulnar nerve and one in the radial nerve, and four cases of stiffness requiring surgical arthrolysis [21]. 

Coronal shear fractures of the distal humerus (type B) pose a substantial challenge as reduction and stabilization are usually demanding [4, 26, 27]. Mighell et al. reported on 18 patients at a mean follow-up of 26 months; the average arc of motion was 128° and the mean Morrey score was 93.3; three patients developed avascular necrosis and five, posttraumatic arthritis [26]. Teng and Zhong reviewed 19 patients with the mean elbow flexion‐extension arc 130.5° and the mean MEPS 85.8. Three patients developed degenerative osteoarthritis and one case heterotopic ossification; a total of 10 patients (53%) underwent removal of implants [27]. Finally, Ashwood et al., in a study of 26 patients who were assessed at mean 46 months postoperatively reported excellent results in nine patients, good in nine, and fair in eight; six patients had altered their roles from manual to administrative work. Three out of six coronal shear fractures treated in our study required revision for implant removal and one followed a rehabilitation program for a longer period due to limited ROM and pain. At the last follow up they achieved satisfying ROM with mean MEPS, OES, and QuickDASH scores of 83.33, 41.17, and 7.58 respectively [4].

The main limitations of our retrospective study are the small size of the cohort, the heterogeneous population with young/old and high/low energy mechanisms, the heterogeneous fracture types, and the wide range of follow-up lengths. The mean follow-up period was 158.16±73.73 months (48-305), which is one of the longest found in the literature. In this long time span, most patients did not seek any postoperative advice so no information regarding medical history in the meantime was available. Moreover, we acknowledge our inability to re-examine all patients at the outpatient department of our institution, because of the imposed restrictive measures due to COVID-19. Our purpose was to analyze the very long-term functional outcome in displaced intra-articular fractures; hence we studied the functional scores and ROM, without assessing radiographic control at the last follow-up.

## Conclusions

We report a small series of surgically treated intra-articular distal humerus fractures that have been followed up for a long time. We achieved a satisfactory range of motion, good functional outcome, and adequate daily activities’ performance. Our results, compared with the ones of similar studies with shorter follow-up, indicate that the outcome of such fractures remains substantially the same after a mean of 13 years. Posttraumatic arthritis, whenever present, can seriously affect functional performance.
